# Revision of Failed Short Stems in Total Hip Arthroplasty

**DOI:** 10.3390/jcm13092459

**Published:** 2024-04-23

**Authors:** Filippo Migliorini, Francesco Coppola, Alessio D’Addona, Marco Rosolani, Federico Della Rocca

**Affiliations:** 1 Department of Orthopaedics and Trauma Surgery, Academic Hospital of Bolzano (SABES-ASDAA), Teaching Hospital of the Paracelsus Medical University, 39100 Bolzano, Italy; 2 Residency Program, University Federico II of Naples, 80131 Naples, Italy; francesco.coppola31@gmail.com; 3 Department of Orthopaedics, Istituto Clinico Humanitas, 20089 Milan, Italy; alessio.daddona@gmail.com (A.D.); marco.rosolani@gmail.com (M.R.); drdellarocca@hotmail.com (F.D.R.)

**Keywords:** total hip arthroplasty, short stem, revision surgery

## Abstract

**Background**: The current literature lacks studies which evaluate the failure of short stems in total hip arthroplasty (THA). Therefore, the present clinical investigation reported our experience with the failure of short stems in THA, evaluating the causes of failure, survivorship, and the clinical outcomes of revision arthroplasty. **Methods**: The present study was performed according to the STROBE guidelines. This study was conducted at the Department of Orthopaedic Surgery of the Humanitas Clinical Institute, Milan, Italy, between 2017 and 2022. All patients who underwent revision surgery of a previously implanted THA using a short stem were prospectively included in the present study. Surgeries were performed with patients in lateral position, using a minimally invasive posterolateral approach. The outcomes of interest were to report information on the type and survivorship of implants used for the revision surgery and evaluate the clinical outcomes and the rate of complications. The following patient-reported outcome measures (PROMs) used for the clinical assessment were the Western Ontario McMaster Osteo-Arthritis Index (WOMAC) and related subscales of pain, stiffness, and function, and the visual analogue scale (VAS). **Results**: Data from 45 patients were retrieved. Of them, 31% (14 of 45 patients) were women. The mean age was 63.7 ± 13.9 years. The mean length of the implant survivorship was 6.2 ± 5.7 years. In total, 58% (26 of 45 patients) underwent revision of all components, 36% (16 of 45 patients) revised only the stem, and 1% (3 of 45 patients) received a two-stage revision. The mean length of the follow-up was 4.4 ± 1.5 years. The cup was revised in 58% (26 of 45) of patients. At 4.4 ± 1.5 years of follow-up, the WOMAC score was 3.5 ± 1.3 and the VAS was 1.2 ± 1.3. In total, 9% (4 of 45) of patients experienced minor complications. One patient used a walking aid because of reduced function. One patient evidenced muscular hypotrophy. Two patients experienced hip dislocations. All two dislocations were managed conservatively with repositioning in the emergency room under fluoroscopy. No patient needed additional revision surgery or experienced further dislocations. **Conclusions**: Revision surgery is effective and safe when a short stem THA fails. At approximately four years of follow-up, all patients were highly satisfied with their clinical outcomes. Despite the relatively high number (9%), complications were of a minor entity and were successfully managed conservatively.

## 1. Introduction

Hip osteoarthritis (OA) is common, with an approximate prevalence of 10% to 30% in the elderly [1,2]. Given the increasing ageing of the population worldwide, the prevalence of OA is increasing [3,4]. Primary OA is multifactorial: both genetics and acquired factors influence the onset of the pathology [5,6]. History of traumas or infections, avascular necrosis of the femoral head, and dysplasia are recognised risk factors for secondary hip OA. Older age and female sex have been associated with a greater risk of hip OA [7,8]. In patients with end stage OA, total hip arthroplasty (THA) might be performed [9,10]. THA aims to restore the quality of life and the participation in recreational activities of patients [6,11]. In recent years, biomaterials, surgical procedures, implants and instrumentations, and management protocols have remarkably improved. In the past two decades, short stem THA has been introduced [12,13]. Several short stems have been designed, including mid-short stem (engaging metaphysis and junction of the metaphysis and diaphysis), and ultra-short stem (engaging only metaphysis) [14,15]. The classification of short stems is controversial, and several classifications have been developed, considering the length, location of loading, osteotomy level, and implant fixation [16,17,18]. Coxa vara, marked anteversion, and older age are contraindications for short stem THA. Indeed, short stems are reserved for young and sport-demanding patients [19,20,21]. The current literature lacks clinical trials investigating recently introduced anatomic short stems or the potential application of cemented short stems. In addition, the current literature lacks investigations evaluating the failure of short stems THA. Therefore, the present study reported our experience with the failure of short stems in THA, evaluating the causes of failure, survivorship, and the clinical outcomes of revision arthroplasty.

## 2. Materials and Methods

The present study was performed according to the Strengthening the Reporting of Observational Studies in Epidemiology (STROBE) [22]. This study was conducted at the Department of Orthopaedic Surgery of the Humanitas Clinical Institute, Milan, Italy, between 2017 and 2022. All patients who underwent revision surgery of a previously implanted THA using a short stem were prospectively included in the present study. The present study was approved and registered by the Ethics Committee of the Humanitas Research Hospital (Protocol Number 618/17) and conducted according to the principles expressed in the Declaration of Helsinki [23]. All patients were able to understand the nature of their treatment and provided written consent to use their clinical and imaging data for research purposes.

### 2.1. Eligibility Criteria

**Figure 1:**
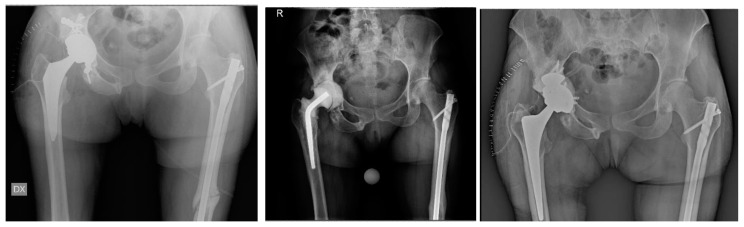
A 47-year-old male patient. Two-stage revision after periprosthetic joint infection three years after THA using GTS (Zimmer Biomet) stem (**left**). Postoperative imaging using an antibiotic-loaded cemented spacer (**middle**). Postoperative imaging using a CLS (Zimmer Biomet) stem (**right**).

**Figure 2:**
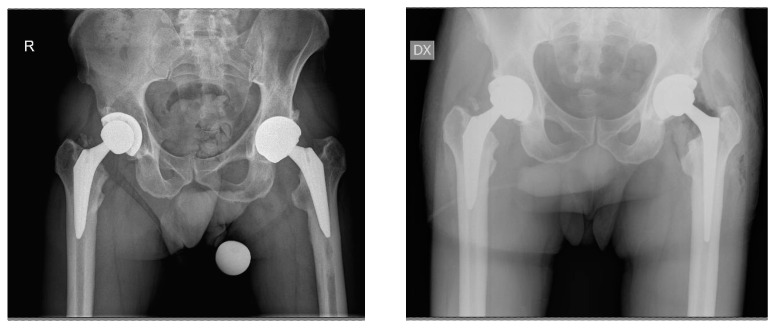
A 56-year-old male patient. Preoperative imaging demonstrating aseptic loosening of the GTS (Zimmer Biomet) stem three years after implantation (**left**). Postoperative imaging using a Wagner Conus (Zimmer Biomet) stem (**right**).

**Figure 3:**
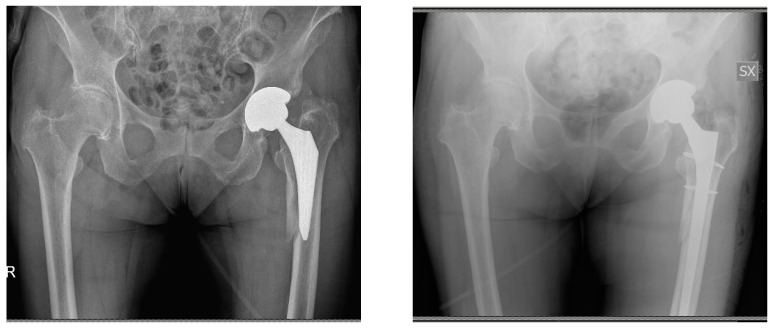
An 85-year-old female patient. Periprosthetic fracture of the GTS (Zimmer Biomet) stem two years after implantation (**left**). Postoperative x-ray after revision surgery with Wagner Revision stem (Zimmer Biomet) implant (**right**).

**Figure 4:**
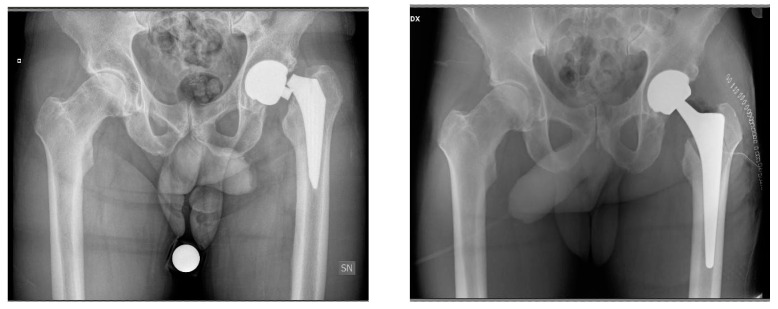
A 55-year-old female patient. Preoperative imaging evidencing rupture GTS (Zimmer Biomet) stem three years after implantation (**left**). Postoperative imaging after stem revision using a CLS (Zimmer Biomet) stem implant (**right**).

**Figure 5:**
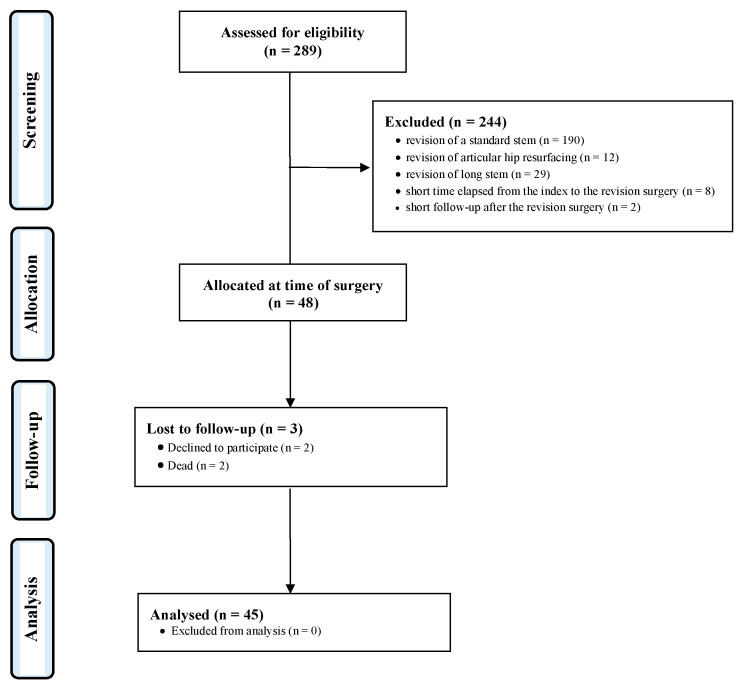
Diagram of the recruitment process.

The inclusion criteria were as follows: (1) partial or total hip revision of a previously implanted short stem, (2) septic or aseptic loosening (Figure 1 and Figure 2, respectively), (3) periprosthetic fracture (Figure 3), (4) stem breaking (Figure 4), (5) minimum of two years elapsed from the index to revision surgery, and (6) minimum two years of follow-up. The exclusion criteria were as follows: (1) isolated acetabular component revision failure postoperatively, (2) revision of a standard stem, (3) revision of a hip resurfacing, (4) revision of a long stem, and (5) patients who declined to participate.

### 2.2. Surgical Technique

The femoral bone stock in preoperative radiographs was assessed using the Paprosky classification [24]. A two-dimensional preoperative planning using Hip Arthroplasty Templating Plugin version 2.4.3 of the OsiriX software version 5.8.1 (Pixmeo SARL, Bernex, Switzerland) was made in all patients. Surgeries were performed with patients in the lateral position, using a minimally invasive posterolateral approach. Before removal, the stability of the primary implant (stem and cup components) was intraoperatively checked and confirmed. The femoral component was removed in all patients. No patients required trochanteric osteotomies or femoral windows to remove the stem. A knife saw was used in the proximal region around the stem to prepare the implant–bone interface. After that, the stem was removed using a dedicated extractor per manufacturer instructions. If the acetabular component was loosed, it was removed from the bone socket. A G7 Acetabular System or Trabecular Metal Modular Acetabular System (Zimmer-Biomet) was used in all patients who required complete THA revision. If the acetabular component was stable, only the liner was changed. The femoral canal is prepared with appropriate instruments of different stems following the two-dimensional preoperative planning. To match the inclination of the acetabular component with femoral anteversion, the femur first technique was always used in all patients [25]. Metal cerclages were used only in cases of revision for periprosthetic fracture. A two-stage revision surgery was used as standard in all periprosthetic joint infections with an antibiotic-loaded cemented hip spacer. No drainage was used.

All patients remained hospitalized for one week. Physiotherapy started on the first postoperative day. Partial weight bearing for the first 6 weeks was allowed. From the 6th week, a gradual increase in partial weight-bearing to full weight-bearing was recommended. All patients received antibiotic prophylaxis on the day of surgery. Patients treated with the two-stage technique for periprosthetic infection used a specific and prolonged antibiotic therapy following recommendations of the infectious disease specialist. Rivaroxaban 10 mg was used for the prophylaxis of the thromboembolism. Naproxen 500 mg was used for the prophylaxis of heterotopic ossification.

### 2.3. Data Collection

On admission, age and sex were recorded. The time elapsed from the index to revision surgery was collected. The types of implants used in the index surgery were retrieved. The protocol and types of revised implants were also retrieved. At the follow-up, patients were telephonically contacted. The outcomes of interest were to report information on the type and survivorship of implants used for the revision surgery and evaluate the clinical outcomes and the rate of complications. The following patient-reported outcome measures (PROMs) used for the clinical assessment were the Western Ontario McMaster Osteo-Arthritis Index (WOMAC) and related subscales pain, stiffness, and function [26], and the visual analogue scale (VAS) [27]. Data on complications were also collected. Data were extracted in Microsoft Office Excel version 16.72 (Microsoft Corporation, Redmond, WA, USA). Concerning the WOMAC score, 24 health-specific items covering pain (five items), stiffness (two items), and function (17 items) were assessed. The subscale scores for pain, stiffness, and function were summed to produce the total score. The pain subscale ranged from 0 (least pain) to 20 (highest pain), stiffness from 0 (least stiffness) to 8 (greatest stiffness), and function from 0 (best function) to 68 (worst function). The final value ranged from 0 (best health) to 96 (worst health). Concerning the VAS, the final value ranged from 0 (no pain) to 10 (worst pain).

### 2.4. Statistical Analysis

All statistical analyses were performed by the main author (FM). The software IBM SPSS version 25 was used. For descriptive statistics, continuous data were shown using the arithmetic mean and standard deviation. Dichotomic data were shown in percentage (number of events/observations). The analysis of PROMs was evaluated using arithmetic mean and standard deviation. The rate of complication was shown in percentage (number of events/observations).

## 3. Results

### 3.1. Recruitment Process

Data from 289 procedures were retrieved. A total of 244 procedures were excluded for the following reasons: revision of a standard stem (*n* = 190), revision of articular hip resurfacing (*n* = 12), revision of long stem (*n* = 29), a short time elapsed from the index to the revision surgery (*n* = 8), and short follow-up after the revision surgery (*n* = 2). This left 48 patients for inclusion. At the follow-up, three patients were lost for the following reasons: declined to participate (*n* = 2) and died (*n* = 1). Finally, 45 patients were included in the present investigation (Figure 5).

### 3.2. Patient Demographics

Data from 45 patients were retrieved. Of them, 31% (14 of 45 patients) were women. The mean age was 63.7 ± 13.9 years. The mean length of the implant survivorship was 6.2 ± 5.7 years. A total of 58% (26 of 45 patients) underwent revision of all components, 36% (16 of 45 patients) revised only the stem, and 1% (3 of 45 patients) received a two-stage revision. The mean length of the follow-up was 4.4 ± 1.5 years. The cup was revised in 58% (26 of 45) of patients.

### 3.3. Results Syntheses

Information on the type of failed implants and used components for revision surgery are reported in Table 1 and Table 2, respectively.

At 4.4 ± 1.5 years of follow-up, the WOMAC score was 3.5 ± 1.3 (Table 3) and the VAS was 1.2 ± 1.3.

9% (4 of 45) of patients experienced minor complications. One patient used a walking aid because of reduced function. One patient evidenced muscular hypotrophy. Two patients experienced hip dislocations. All two dislocations were managed conservatively with repositioning in the emergency room under fluoroscopy. No patient needed additional revision surgery or experienced further dislocation.

## 4. Discussion

According to the main findings of the present clinical investigation, revision surgery is effective and safe when a short stem THA fails. At approximately four years of follow-up, PROMs indicated a very good clinical and functional outcome. Despite the relatively high number (9%), complications were of a minor entity and were successfully managed conservatively.

Short stems are among the successful implants in terms of early-stage survivorship. In the present investigation, aseptic stem loosening appeared in the short term and was caused by a varus mispositioning or undersizing of the component. In the German national joint registry, short stems, such as the Nanos stem, the A2 stem, the MiniHip stem, and the Optimys stem, have been associated with excellent implant survival [28]. These results are similar to those reported in the Australian and Swiss national registries [29,30]. However, only mid-term registry data are currently available. A recent meta-analysis compared short versus conventional stems in THA [31]. Short stem THA could be associated with greater patient-reported outcome measures (PROMs) and lower blood loss. No difference was found in leg length discrepancy and complications. Short stem THA is of special interest to younger patients. Short stem THA preserves more bone stock, thus providing favourable conditions for further revisions. The procedure of implantation of a short stem requires smaller surgical exposure, which might reduce soft tissue damage, simplifying a minimally invasive approach and providing better aesthetic outcomes. Given their reduced length in the major axis, short stems can be changed using conventional stems in revision settings.

**Figure 6:**
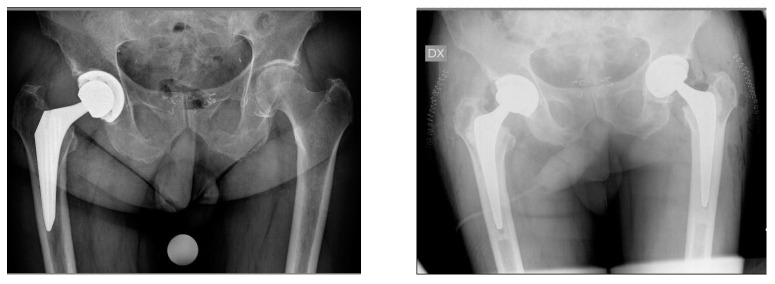
A 77-year-oldmale patient. Preoperative imaging demonstrating aseptic loosening of a GTS (Zimmer Biomet) stem two years postoperatively (**left**). Postoperative imaging after stem revision with MS-30 (Zimmer Biomet) cemented stem ipsilateral and primary contralateral THA using an MS-30 (Zimmer Biomet) cemented stem (**right**).

Stress shielding in cementless THA is common and is a cause of complications. The metallic stem absorbs part of the weight-bearing stress of the bone, reducing bony stress and leading to demineralisation. The metallic stiffness, along with the impaired load transmission, increases the stress shielding, increasing bony resorption and lateral and distal bony ingrowth. Moreover, conventional stems might promote cortical hypertrophy at diaphysis around the distal stem which might create a mechanical impingement with the stem itself and bony compression. This remodelling might be associated with thigh pain. Given their engagement at the proximal femur, short stems shall avoid stress shielding and distal stem cortical hypertrophy. Increased stress shielding might increase bone resorption, aseptic loosening (Figure 6), fracture, and revision. The recently published literature demonstrated that a combination of a short and an anatomically designed stem with a low stiffness might provide an additional physiological strain transfer during THA, reducing the risk of stress shielding [32].

A formal preoperative assessment of PROMs was not conducted, as all patients were symptomatic and presented with loss of function at admission. Given the lower number of procedures considered, along with the heterogeneity in the used implants in the primary and revision THAs and the variability of the causes of revision, a formal subgroup analysis was not possible. Moreover, given the limited sample size, it was not possible to establish whether certain causes of revisions (e.g., infection or periprosthetic fractures) might negatively impact the outcome. Further clinical investigations should evaluate the outcomes on a larger scale.

## 5. Conclusions

Revision surgery is effective and safe when a short stem THA fails. At approximately four years of follow-up, all patients were highly satisfied with their clinical outcomes. Despite the relatively high number (9%), complications were of a minor entity and were successfully managed conservatively.

## Figures and Tables

**Table 1:** Type and cause of failed short stems (other: malpositioning error or stem breakage).

Type of Implant	Patients (*n*)	Mean Implant Survivorship (years)	Aseptic Loosening (*n*)	Septic Loosening (*n*)	Fracture (*n*)	Other (*n*)
GTS (Zimmer Biomet)	20	3.8	15	1	3	3
FITMORE (Zimmer Biomet)	6	2.8	4	1		1
CFP (Waldemar Link)	6	14.2	6			
NANOS (Smith & Nephew)	1	5.0	1			
TAPERLOC (Zimmer Biomet)	1	9.0	0			1
Other less commonly used stems	11	7.8	10	1		1

**Table 2:** Type of implants used for the revision with related data on Paprosky classification, length of the follow-up, and causes for revision (FU: follow-up).

Type of Implant	Patients (*n*)	Paprosky	Mean FU (years)	Mean age	Fracture (*n*)	Septic Loosening (*n*)	Aseptic Loosening (*n*)	Other (*n*)
CLS (Zimmer Biomet)	12	I–II	4.2	56.0		2	9	3
GTS (Zimmer Biomet)	2	I–II	4.5	69.5			2	
MS-30 (Zimmer Biomet)	3	I–IV	4.0	70.6	1		2	
PYRAMID (Zimmer Biomet)	2	I–II	2.5	65.5			2	
WAGNER KONUS (Zimmer Biomet)	5	I–III	5.2	54.8		1	4	
WAGNER REVISION 190/225 (Zimmer Biomet)	18	I–IV	4.4	58.6	2		16	
ARCOS (Zimmer Biomet)	3	II–IV	5.3	69.9			3	

**Table 3:** Results of the WOMAC score and related subscales.

WOMAC	
Pain	0.6 ± 0.1
Stiffness	0.2 ± 0.1
Function	2.6 ± 0.1
Total	3.5 ± 1.3

## Data Availability

The data presented in this study are available on request from the corresponding author.
